# Going Commando as Part of a Multifaceted Intervention to Reduce CAUTIs in Critically Ill Children

**DOI:** 10.1017/ash.2024.307

**Published:** 2024-09-16

**Authors:** Matt Linam, Lisette Wannemacher, Kathryn Powell, Christina Calamaro, Karen Walson

**Affiliations:** Emory University; Children’s Healthcare of Atlanta

## Abstract

**Background and Objectives:** Catheter associated urinary tract infections (CAUTIs) are a source of preventable harm in children. Insertion and maintenance bundles have significantly reduced CAUTIs, but infections still occur. Starting in mid-2019, we experienced an increase in CAUTIs in our pediatric intensive care unit (PICU). The objective was to identify preventable causes of CAUTI and develop and test interventions to reduce them. **Methods:** This quality improvement project was initiated in the PICU of a large tertiary children’s hospital. Interdisciplinary rounds led by the hospital epidemiologist and unit nursing leader with the bedside nurse occurred weekly (starting October 2019) for patients with urinary catheters in place for greater than three days. Discussions included strategies to optimize maintenance of the urinary catheter and identify catheters that could be removed. Additional interventions included no diapers for patients with a urinary catheter (starting March 2021) and use of a urine collection device that prevented both urine stasis in the drainage tube and retrograde flow of urine into the bladder (starting August 2021). Hand hygiene and CAUTI prevention bundle compliance was measured by direct observation of staff. CAUTIs were identified by prospective surveillance by infection prevention using standard definitions. The rate of CAUTIs over time was analyzed using statistical process control charts. **Results:** The baseline CAUTI rate (January 2017 - June 2019) was 0.5 infections/1000 catheter days with an average of 349 days between CAUTIs. Between July 2019 and February 2021, the CAUTI rate increased to 3.3 with an average of 88 days between CAUTIs. Annual compliance with hand hygiene and the CAUTI prevention bundle elements remained above 90% throughout all time periods. No improvement was seen after the institution of weekly interdisciplinary rounds. Starting in March 2021 after removal of diapers and implementation of the urine collection device that prevented retrograde flow, the CAUTI rate decreased to 0.9 and an average of 200 days between CAUTIs. Currently, it has been 512 days since the last CAUTI. **Conclusion:** CAUTIs decreased after removing diapers in children with urinary catheters and use of the urine collection device. Removal of diapers likely reduced stool contamination around the catheter and urethral opening. The urine collection device prevented inadvertent retrograde flow of urine into the bladder. These interventions could augment current CAUTI prevention strategies.

